# Quantifying the effects of biopsy fixation and staining panel design on automatic instance segmentation of immune cells in human lupus nephritis

**DOI:** 10.1117/1.JBO.26.2.022910

**Published:** 2021-01-08

**Authors:** Madeleine S. Durkee, Rebecca Abraham, Junting Ai, Margaret Veselits, Marcus R. Clark, Maryellen L. Giger

**Affiliations:** a University of Chicago, Committee on Medical Physics, Department of Radiology, Chicago, Illinois, United States; b University of Chicago, Section of Rheumatology and Gwen Knapp Center for Lupus and Immunology Research, Department of Medicine, Chicago, Illinois, United States

**Keywords:** instance segmentation, high-throughput image analysis, deep learning, immunology

## Abstract

**Significance:** Lupus nephritis (LuN) is a chronic inflammatory kidney disease. The cellular mechanisms by which LuN progresses to kidney failure are poorly characterized. Automated instance segmentation of immune cells in immunofluorescence images of LuN can probe these cellular interactions.

**Aim:** Our specific goal is to quantify how sample fixation and staining panel design impact automated instance segmentation and characterization of immune cells.

**Approach:** Convolutional neural networks (CNNs) were trained to segment immune cells in fluorescence confocal images of LuN biopsies. Three datasets were used to probe the effects of fixation methods on cell features and the effects of one-marker versus two-marker per cell staining panels on CNN performance.

**Results:** Networks trained for multi-class instance segmentation on fresh-frozen and formalin-fixed, paraffin-embedded (FFPE) samples stained with a two-marker panel had sensitivities of 0.87 and 0.91 and specificities of 0.82 and 0.88, respectively. Training on samples with a one-marker panel reduced sensitivity (0.72). Cell size and intercellular distances were significantly smaller in FFPE samples compared to fresh frozen (Kolmogorov–Smirnov, p≪0.0001).

**Conclusions**: Fixation method significantly reduces cell size and intercellular distances in LuN biopsies. The use of two markers to identify cell subsets showed improved CNN sensitivity relative to using a single marker.

## 1 Introduction

### 1.1 Clinical Motivation: Lupus Nephritis

Lupus nephritis (LuN) is a chronic inflammatory kidney disease that is a manifestation of systemic lupus erythematosus (SLE). It is characterized by immune-mediated kidney damage driven by both the damage from self-reactive antibodies and the infiltration of the kidney by various cell types (e.g., lymphocytes and dendritic cells) that comprise the immune system. Over the course of the disease, this damage accumulates and can result in kidney failure or end stage renal disease (ESRD). This requires either dialysis or transplant, resulting in substantial morbidity and mortality for the approximately 40% of SLE patients that present with LuN.^1^^,^^2^ Less than 60% of patients with severe LuN will respond to current treatment protocols.^3^ Kidney biopsies are a critical tool for diagnosing and grading LuN.^4^ One metric of disease severity is tubulointerstitial inflammation (TII), which quantifies the infiltration of CD45+ immune cells into the kidney. Notably, nearly half of patients with high TII score will progress to ESRD within 4 years.^5^^,^^6^

While large-scale evaluations of the spatial distribution of inflammation within the kidney can be made, robust methods for characterizing that inflammation in terms of what cell types are present and how they interact with each other are lacking. Because intercellular interactions are at the core of all immunological phenomena, it is difficult to understand the inflammatory processes that are taking place within inflamed tissue without granular spatial information. A consequence of this is that several treatment modalities that aim to address these immunological processes have failed to live up to their promise.^7^^–^^9^

We previously demonstrated that multi-channel fluorescence confocal microscopy can be used in conjunction with computer vision techniques to investigate the interactions between different populations of lymphocytes within LuN biopsies.^10^^,^^11^ In this context, computer vision allows for automated detection of immune cells in inflamed tissue, which will help to improve understanding of autoimmune phenomena in diseases such as LuN. However, applying computer vision to segment immune cells in inflamed human tissue remains a challenging task, due to issues such as tissue autofluorescence and variable antibody uptake. Therefore, it is important to understand how aspects of data collection impact the performance of computer vision applications. Here, we evaluate the robustness of these methods to automatically assess cell prevalence and shape in three separate datasets of LuN biopsies. Our goal is to inform decision-making in future data collection so that we can use these techniques to further our understanding of inflammatory disease.

### 1.2 Deep Learning in Cellular Images

High-throughput analysis of cellular imaging is a difficult and time-consuming task. Specifically, there is no effective and efficient manual method for reliably quantifying cell location and shape, a task that is important for understanding intercellular interactions. Since the emergence of deep learning as a state-of-the-art computer vision technique, it has become an integral tool in the identification, segmentation, and classification of cells and cell nuclei in microscopy images.^12^ For diagnosis and grading of pathology slides, deep learning models have shown high accuracy in rapid classification of slides. Additionally, deep learning models currently outperform other automatic segmentation methods in most tasks involving the segmentation of cell nuclei. However, in dense aggregates of cells and multi-class images, individual cell classification and segmentation remain difficult tasks.^13^

Multiple deep convolutional neural network (CNN) architectures have been developed to improve automatic instance segmentation of cells, a computer vision task that identifies, segments, and classifies multi-object, multi-class images of cells. Multiple CNN architectures have shown promising results in the task of instance segmentation of cell nuclei in fluorescence images. In general, segmentation architectures, such as the U-Net, and region-based methods, such as mask R-CNN, are commonly used or adapted to accurately segment cells in various modalities of microscopy images.^14^^,^^15^ Narotamo et al.^16^ combined fast YOLO—an object detection network architecture—with a U-Net to segment individual nuclei in images while minimizing computational complexity. Network architectures have also been combined into ensembles or cascades to improve performance for a given computer vision task. A mask R-CNN and U-Net ensemble network was trained to segment cell nuclei in images from multiple modalities, bright-field, fluorescence, and RGB wide-field histology.^17^ Unlike these examples, the task at hand requires multi-class instance segmentation, and we need to be more specific than nuclear segmentation as each class of cells is defined by the nuclear marker plus one or more immunological markers. Liarski et al.^10^ developed a custom network for this task. Here, we implement mask R-CNN^18^ to segment and classify immune cells in three different datasets of fluorescence confocal images of human LuN biopsies. Mask R-CNN is computationally expensive but has been shown to yield high accuracy for multi-class instance segmentation, so we have adapted this architecture to work with 6-channel fluorescence confocal images to segment three to five classes of cells in each image.

### 1.3 Dataset Variability and Probing *in situ* Immunity in LuN

The cellular markers investigated in this study were selected in order to investigate the interactions between CD4+ T cells, CD4− T cells, and potential antigen presenting subsets in LuN, including B cells, plasmacytoid dendritic cells (pDCs), and myeloid dendritic cells (mDCs). Previous work in this field has shown that these cell types might play a role in the pathogenesis of LuN.^5^^,^^10^ B cells have long been appreciated for their role in lupus, as it is an antibody-mediated disease and B cells are antibody-producing cells. T cells make up a large proportion of the infiltrating immune cells in this disease,^19^ and it is thought that they might modulate disease progression both by providing “help” to the other infiltrating immune cells and by directly acting on the tissue. Dendritic cells modulate the activity of T cells by presenting antigen to them, leading to either their activation of suppression, depending on the context.^4^ Understanding the complex interplay of these cell populations is therefore of great interest and motivates the development of computer vision techniques for this purpose.

Using clinical samples is resource intensive, so it is vital to optimize data collection for the chosen analytical method. Specifically, it is important to understand how technical choices regarding sample preparation might influence the quality of data used in automated cell detection algorithms.

There are two major considerations we wish to address here. First, does the method of sample preparation influence our findings around cellular morphology? Our previous work was performed on fresh-frozen samples. These are relatively expensive to store, and far less widely available than formalin-fixed, paraffin-embedded (FFPE) tissue. Extending this technique to FFPE samples would greatly increase the dataset of samples that are available. However, it is well-established that formalin fixation can lead to gross tissue shrinkage,^20^^–^^22^ which could lead to distortions in our findings around cell shape, size, and distance to other cell types. In this work, we seek to understand whether these deformations cause differences in cell shape and intercellular distances. Given no statistical difference between these two groups, it would be appropriate to group fresh-frozen biopsies and FFPE biopsies for analysis of cellular features, which would increase the availability of datasets. For this reason, we evaluated performance of independently trained mask R-CNN networks in the task of multi-class instance segmentation of cells in FFPE tissue samples relative to fresh frozen.

Second, we wanted to address the number of stains that are required to identify a cell type. Our previous work utilized two markers to identify each type of dendritic cell. However, the ability of a given microscope to resolve adjacent emission spectra limits the number of fluorophores that are available for a staining panel to 5 or 6 markers. To the human observer, using multiple markers to identify a cellular class results in better discrimination of cell classes. In manual analysis of cells, this approach helps to identify true positives because tissue autofluorescence, stain quality, spectral bleed-through, and non-specific antibody binding can result in ambiguous signal. However, due to the limitation in the number of fluorophores that can be resolved in one imaging session, the choice to use multiple markers per cell type necessarily means that fewer cell types can be investigated in a given panel. This is essentially a trade-off between robustness and breadth. Here, we investigate whether single markers can be used in a computer vision task to identify and segment cell types with high fidelity, allowing us to expand the set of cell types we examine with a single panel.

For these two purposes, we collected three datasets from kidney biopsies of LuN patients: (1) fresh frozen, stained with two markers per antigen presenting cell (APC), (2) FFPE, stained with two markers per APC, and (3) FFPE, stained with one marker per APC. These findings will allow us to optimize future data collection efforts for the application of computer vision, which will enable rigorous quantification of immune cell subsets in tissue.

## 2 Methods

### 2.1 Data Acquisition

For staining of fresh-frozen sections, the sections were removed from −80°C, washed with PBS, blocked with serum and followed by antibody staining. Two distinct antibody panels were utilized to stain the tissue sections: for pDC analysis—CD3 (Alexa Flour 546), CD4 (Alexa Flour 594), BDCA2 (Alexa Flour 488), and CD123 (Alexa Flour 647); mDC analysis—CD3 (Alexa Flour488), BDCA1 (Alexa Flour546), CD4 (Alexa Flour594), and CD11c (Alexa Flour647). 4′,6-diamidino-2-phenylindole (DAPI) (Hoechst 33342, Invitrogen) was used with the above to visualize tissue nuclei. Fresh-frozen tonsil sections served as controls. For staining of FFPE sections, the sections were de-paraffinized, treated with citric acid buffer (pH 6.0) for antigen retrieval, then blocked and stained with the same process of fresh-frozen samples. Double staining on FFPE (FFPE-DS) was done with the same fluorophores as fresh frozen. Single staining (FFPE-SS) was done with CD20 (Alexa Flour 488), CD3 (Alexa Flour546), BDCA2 (Alexa Flour594), CD4 (Alexa Flour647), and CD11c (Alexa Flour700). FFPE tonsil sections served as controls. Further details on selected antibodies are listed in Table S1 in the Supplementary Material.

### 2.2 Lupus Nephritis Datasets

**Fig. 1:**
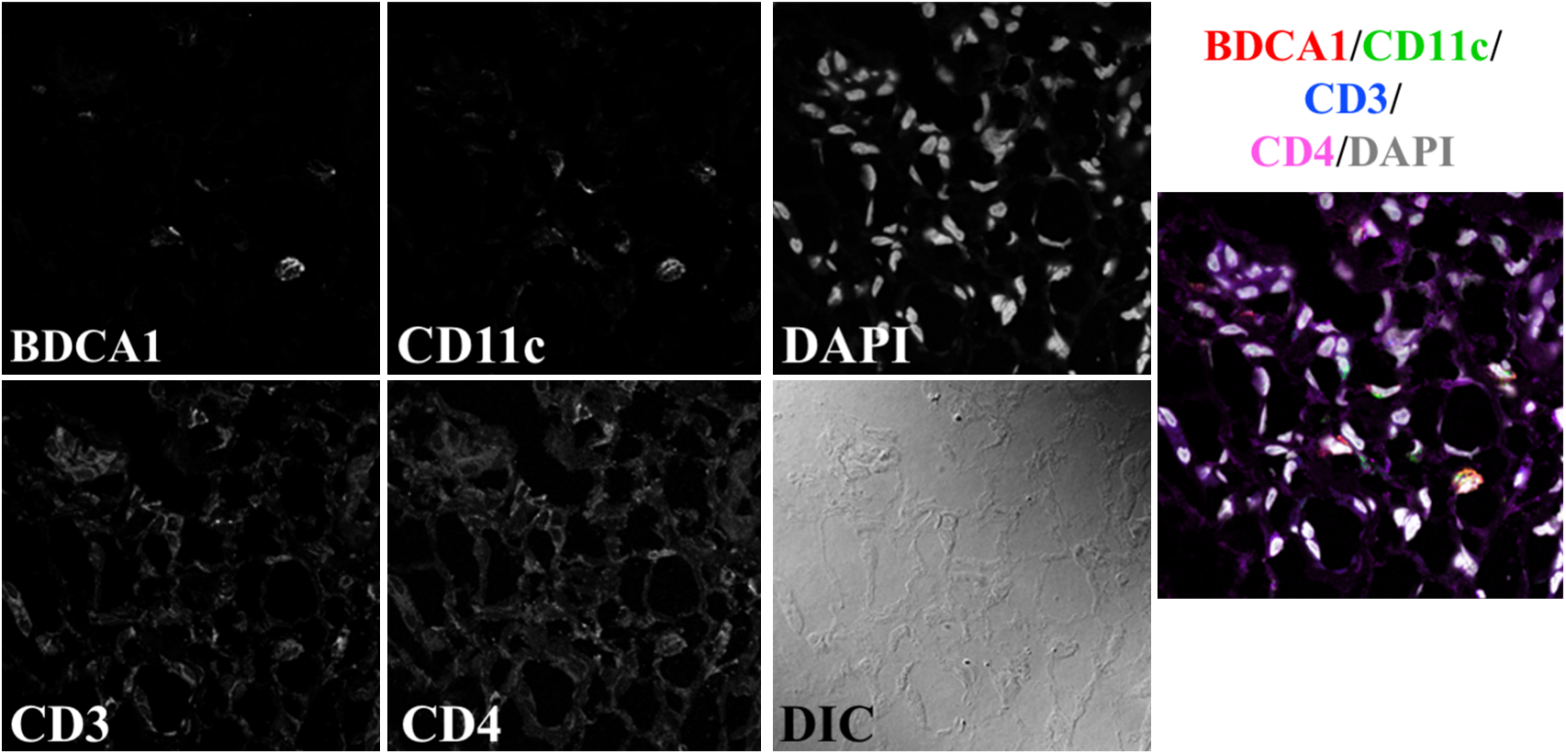
Example image from the fresh-frozen-DS dataset. A single-APC population (mDCs) is probed with two markers. This example shows a mDC image, but pDC images are also in this dataset, with the markers listed in Table 2. T cells are stained for CD3 and CD4, and DAPI is used to identify cell nuclei. All channels are merged in the rightmost panel, with colors corresponding to the above label.

Three separate LuN datasets were used in this study to compare two tissue fixation methods (fresh frozen and FFPE) and two staining panels (Table 1). The first dataset (fresh-frozen-DS) was composed of images of fresh-frozen LuN biopsies, imaged on a Leica SP5 laser scanning confocal microscope at 63× magnification. Resulting images were 1024×1024  pixels with a 0.1413-μm pixel size (Table 1). The samples in this dataset were stained with staining panel 1 (Table 2), using two markers per APC. As a result, a given sample was only stained for two T cell populations and one APC population, either mDCs or pDCs, and each image consisted of three cell classes: CD3+CD4+ T cells, CD3+CD4− T cells, and one type of dendritic cell (Fig. 1).

**Table 1:** Defining descriptors of the three datasets used to assess DCNN performance on fixation methods and staining panels.

	Fixation method	Staining panel	Microscope	Pixel size (μm)
Fresh-frozen-DS	Fresh frozen	Panel 1	Leica SP5	0.1337 to 0.1413
FFPE-DS	FFPE	Panel 1	Leica SP8	0.1058
FFPE-SS	FFPE	Panel 2	Leica SP8	0.1058

**Table 2:** Two staining panels were used to compare DCNN performance on single- to dual-marker identification of APCs.

	T cell markers	mDC markers	pDC markers	B cell markers	Nuclear marker	Other
Panel 1 (DS)	CD3, CD4	CD11c, BDCA1	BDCA2, CD123	—	DAPI	DIC
Panel 2 (SS)	CD3, CD4	CD11c	BDCA2	CD20	DAPI	—

**Fig. 2:**
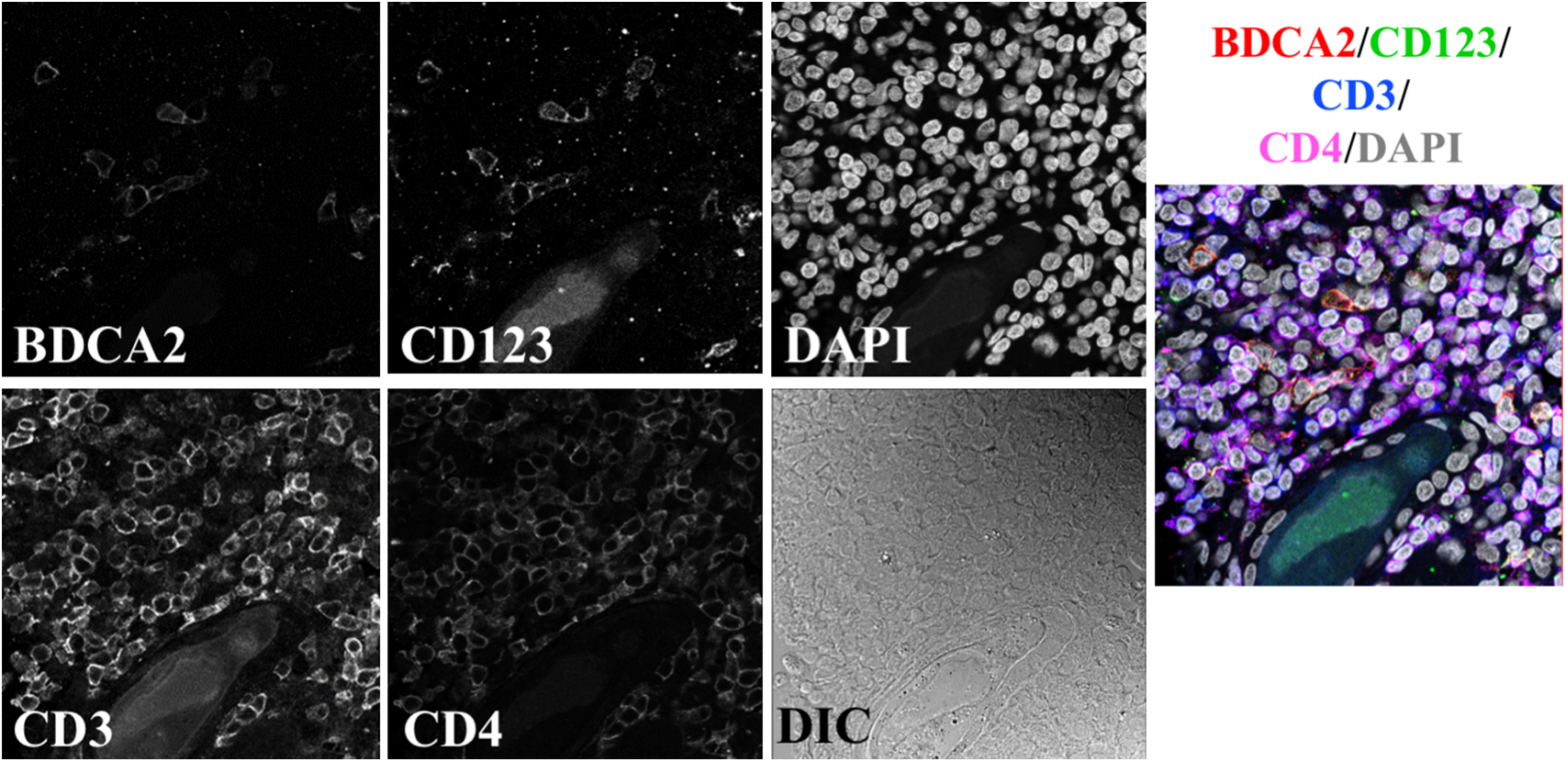
Example image from the FFPE-DS dataset. A single-APC population (pDCs) is probed with two markers. This example shows a pDC image, but mDC images are also in this dataset, with the markers listed in Table 2. T cells are stained for CD3 and CD4, and DAPI is used to identify cell nuclei. All channels are merged in the rightmost panel, with colors corresponding to the above label.

**Fig. 3:**
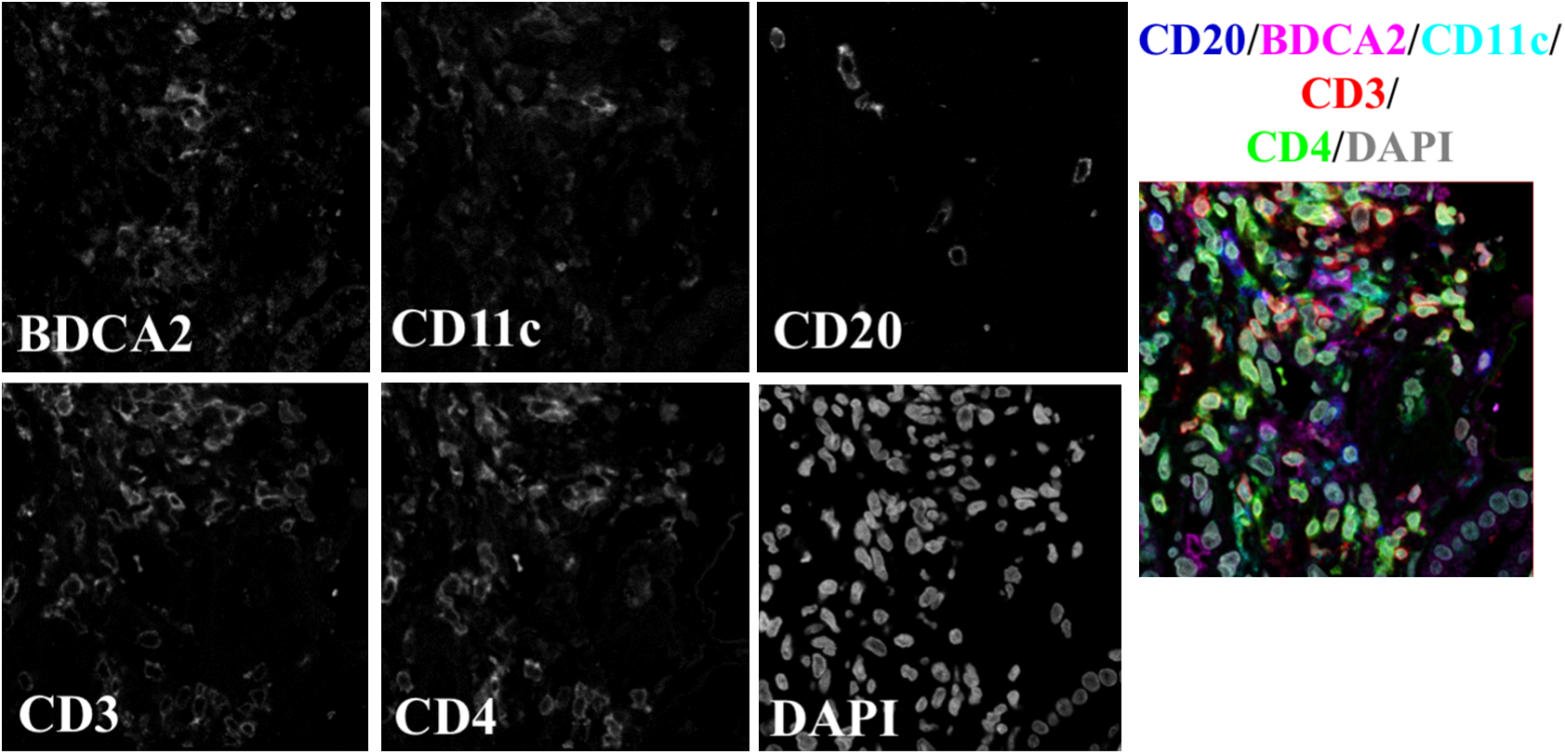
Example image from the FFPE-SS dataset. Three APC populations (mDCs, pDCs, and B cells) are probed with a single marker each. T cells are stained for CD3 and CD4, and DAPI is used to identify cell nuclei. All channels are merged in the rightmost panel, with colors corresponding to the above label.

FFPE-DS and FFPE-SS datasets were composed of images of FFPE samples, imaged on a Leica SP8 laser scanning confocal microscope at 63× magnification. Images remained 1024×1024  pixels; however, given the different imaging system, the resulting pixel size for these two datasets is 0.1058  μm. The FFPE-DS dataset was also stained with panel 1, with three cell classes per image (Fig. 2). The FFPE-SS dataset was stained with a single marker per APC class. In addition to staining for both pDCs and mDCs in one panel, B cells were also probed in this dataset, resulting in five cell classes: two T cell populations and three APC populations (Fig. 3). The FFPE-SS dataset lacks a differential interference contrast (DIC) channel in order to accommodate an additional cell surface marker while maintaining a constant channel depth. Conservation of channel depth was desirable for this study because keeping this variable consistent preserves the number of trainable parameters in the network. In the DS datasets, the DIC channel was intended to aid in the segmentation of cells, as it mainly contributes cell edge information. Preliminary analysis of the FFPE-SS dataset determined that the pixel-level segmentation was not adversely affected by eliminating this channel. Resulting image stacks were 1024  pixels×1024  pixels × 6  channels, with each channel associated with a single marker. Table 2 summarizes the key differences in the three datasets.

### 2.3 Manual Segmentation of Images for Ground Truth

For all datasets, a subset of images from each biopsy was selected on which to generate manual truth. All manual segmentations and cell classifications were done using Fiji/ImageJ software to generate free-hand outlines of the cells. For all three datasets, the ground truth was established through two rounds of segmentation. The first round was performed by several researchers with experience with evaluating microscopy data. The second round for all three sets was done by a single final expert, a researcher in a rheumatology lab with extensive experience in reading and analyzing multi-channel immunofluorescence images. Instructions for generating truth were to outline each cell-based off of the surface marker(s) that defined each class, given the constraint that nuclear signal was present in the DAPI channel within this outline. The second round of segmentation by one expert was conducted to address the issue of reader fatigue and maintain consistency. Because we were interested in how various aspects of data acquisition affected performance, and we wanted to avoid the confounding variable of inter-observer variation. Cell identification, classification, and pixelwise segmentation were all performed manually, such that no automation was included in generating ground truth. In the FFPE-SS dataset, the higher number of channels resulted in greater spectral overlap between fluorophores, and some manual classifications became ambiguous. To aid the generation of manual truth, the channels were spectrally unmixed using the excitation and emission spectra of each fluorophore. The experts were given the spectrally unmixed images to determine ground truth, but network training used the raw image data.

### 2.4 Generation of Training Sets

Manually segmented images were split into training, validation, and test sets at a 90/5/5 ratio (Table 3). Validation and test sets were small at the image level, but still contained over 300 cells each, and network performance is measured at the cell level. The FFPE-DS dataset had a smaller ground truth set due to the large number of cells per image in that dataset. The FFPE-SS manual dataset contained more images with a relatively high density of cells. The large number of manually segmented cells in the FFPE-SS manual truth set caused a 90% training set to exceed our GPU memory capabilities (4 nVidia K80 GPUs with 12 GB memory each). The ground truth for this dataset was therefore split into training/validation/testing sets at an 85/7.5/7.5 ratio. Images from a given biopsy were randomly divided up between the training/validation/test sets. This means that, while there were unique sets of images in the training/validation/test sets, images from the same biopsy could be in more than one of these subsets. This was done intentionally for this study to ensure that differences in performance between the separately trained instances of mask R-CNN were due to the staining panel or fixation method, and not differences between patients in the training and testing sets.

**Table 3:** Training, validation, and test set splits for the manual segmentations in all datasets.

	Total cells in manual set	Total images in manual set	Images in training set	Images in validation set	Images in test set
Fresh-frozen-DS	5166	240	168 (90%)	12 (5%)	12 (5%)
FFPE-DS	7145	160	143 (90%)	8(5%)	8 (5%)
FFPE-SS	10611	342	293 (85%)	26 (7.5%)	26 (7.5%)

### 2.5 Network Architecture and Training

**Fig. 4:**
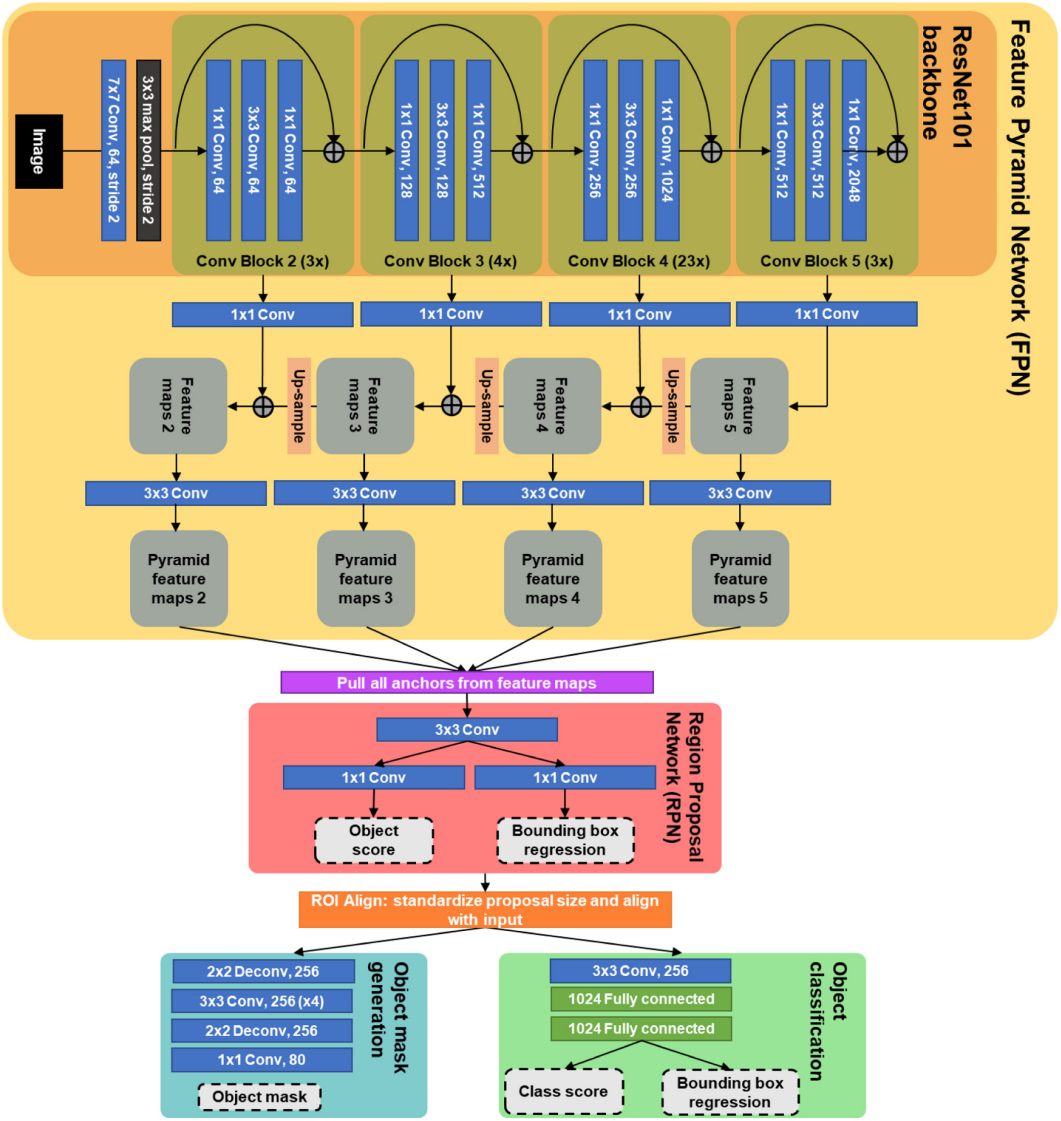
Each network trained to segment and classify immune cells is a mask R-CNN architecture. Object proposals are performed on feature maps from the DCNN, and then single objects (cells) are semantically segmented and classified.

Three separate instances of a mask R-CNN architecture^18^ were trained to conduct instance segmentation on each of the three datasets. Mask R-CNN is part of a family of region-based CNNs that are designed for instance segmentation.^18^^,^^23^^,^^24^ The overall architecture is described in Fig. 4. A feature pyramid network (FPN) is used as a feature extractor. The backbone of this FPN in this paper is a ResNet101 architecture.^25^ The average-pooling layer, fully connected layer, and softmax layer normally found at the end of a ResNet101 are left off, as the network is used to generate feature maps rather than classify full images. In the FPN structure, feature maps are pulled out of the ResNet blocks at all scales and run through a 1×1 convolutional layer. Higher level (lower resolution) feature maps are upsampled and summed with lower level (higher resolution) feature maps. These “multiscale” feature maps are passed through 3×3 convolutional layers in preparation for input into the region proposal network (RPN). Predetermined anchor boxes of various sizes and aspect ratios are pulled from each position of the feature maps for input into the RPN. The RPN is a small network that operates on these anchors to propose objects and is comprised of a single 3×3 convolutional layer and two “sibling” 1×1 convolutional layers for (1) determining whether a given proposal is in fact an object and (2) bounding box regression. The object proposals from this RPN are converted to fixed-size proposals and aligned with feature maps, then each object progresses in parallel through (1) fully connected layers for classification and further bounding box regression and (2) mask generation.

Hyperparameters were tuned to optimize accuracy on multiple class sets. The networks were trained with a learning rate of 0.01 using stochastic gradient descent with momentum. Cells in dense regions were detected with higher accuracy by reducing hyperparameter of the RPN section of the network, anchor stride length. Training was monitored using Tensorboard and was stopped once the mean average recall for all cell classes stopped increasing. A cell was kept for analysis if the network confidence in the prediction was above 0.3.

All image preparation, network training, and inference were performed using the Midway2 compute nodes of the University of Chicago Research Computing Center. Each network was trained separately on each dataset, with a batch size of 4 distributed across 4 Nvidia K80 GPUs (12 GB memory each) using Horovod distributed deep learning framework.^26^ Data augmentation included random flips and rotations, and brightness and gamma augmentation.

### 2.6 Evaluation of DCNN Performance

DCNN performance was measured by calculating sensitivity, specificity, and Jaccard index, also known as intersection over union (IOU), for a test set. The manual segmentations provided ground truth at the cell level. A cell prediction was considered a true positive if it had an IOU of at least 0.25 with a manual segmentation of a cell of the same class. Sensitivity and specificity for cell detection and classification were calculated at the cell class level. In addition, IOUs were calculated on a per cell basis and averaged across all cells within a given cell class. Sensitivity, specificity, and IOU were averaged across all cells to provide overall performance metrics for the networks.

### 2.7 Cell Shape and Distance Metrics

After analyzing the detection and segmentation performance of each network on the corresponding test sets, each network was used to predict cell types in larger sets of unlabeled images. Population ratios of each cell type were calculated for each unlabeled dataset and compared to the corresponding ratios in the ground truth dataset. After analyzing the performance of the three trained networks, each network was used to predict cell types in unlabeled images. Cell size, shape, and distance features, specifically cell area, cell perimeter, and T cell minimum distance to a DC, were calculated for each cell detected by the networks. These shape features were compared across datasets to determine whether the tissue preparation method, stain specificity, or network performance affected cellular features.

## 3 Results

### 3.1 Network Performance on Test Sets

Deep CNNs with mask R-CNN architectures were trained for each of the three separate datasets. Both instances of mask R-CNN trained on the fresh-frozen-DS and FFPE-DS datasets met the stopping criteria at 64k iterations with a batch size of 4 or 16k epochs. Performance metrics on test sets for these two datasets are detailed in Table 4. It is important to note that while DC sensitivity is high, we do not necessarily expect to detect every DC in an unlabeled dataset. These test sets are relatively small at the image level, and DCs are the least prevalent populations, but there are still over 50 DCs in each test set. Each network trained on a double-stain dataset detected all DCs in the corresponding test set, regardless of sample fixation method.

**Table 4:** A network was trained and tested on each dataset as described in Tables 1–3. Sensitivity, specificity, and Jaccard index (IOU) are shown for the test sets corresponding to the two networks trained on the double-stain datasets.

	CD3+CD4+ T cells	CD3+CD4− T cells	DCs	All (average)
Sensitivity				
Fresh-frozen-DS	0.77	0.85	1.0	0.87
FFPE-DS	0.89	0.84	1.0	0.91
Specificity				
Fresh-frozen-DS	0.82	0.84	0.80	0.82
FFPE-DS	0.84	0.83	0.96	0.88
IOU				
Fresh-frozen-DS	0.79±0.21	0.75±0.24	0.83±0.19	0.80±0.21
FFPE-DS	0.77±0.22	0.80±0.19	0.86±0.15	0.79±0.20

The instance of mask R-CNN trained on the FFPE-SS dataset required longer training time (72k iterations or 18k epochs), and network sensitivity was poor for mDCs and marginal for pDCs (Table 5). The poor performance on DCs may be due to the fact that they are more amorphous than lymphocytes such as T cells and B cells, which have relatively little cytoplasm and therefore have surface stains that coincide with their nuclei. In contrast, dendritic cells have long extensions from their cell bodies called dendrites,^27^ which can reach in and out of the image plane, producing positive signal where there may not be a nucleus to assign it to. Therefore, assigning ground truth to these cells is inherently harder. In the fresh-frozen-DS and FFPE-DS datasets, DCs are identified with two markers, whereas in the FFPE-SS dataset, each DC population is identified with a single marker. With this dataset, we tested the hypothesis that using multiple stains to identify DCs bolsters performance, and that using only one marker would impose a cost. The decline of network performance on these cells is likely due to a combination of low signal-to-noise ratio, variable cell shape, and ambiguous ground truth.

**Table 5:** A network was trained on the FFPE-SS dataset. Sensitivity, specificity, and Jaccard index (IOU) are shown for the FFPE-SS test set.

	CD3+CD4+ T cells	CD3+CD4− T cells	mDCs	pDCs	B cells	All cells (average)
Sensitivity FFPE-SS	0.90	0.85	0.38	0.69	0.75	0.72
Specificity FFPE-SS	0.86	0.89	0.97	0.95	0.91	0.92
IOU FFPE-SS	0.81±0.17	0.82±0.18	0.63±0.21	0.74±0.20	0.75±0.21	0.78±0.19

The three trained networks described above were used to generate cell predictions on larger unlabeled datasets. Table 6 describes the manual and automatic segmentations for each of the three datasets. Each trained instance of mask R-CNN was used to generate cell predictions on all images in its corresponding dataset, which included unlabeled versions of all images that had been manually segmented and images that were never manually segmented by an expert. The average number of cells per image is similar between the manual segmentations and automatic predictions for each dataset. Assuming patients in these larger datasets have similar prevalence of each cell type, the manual and automatic segmentation sets should maintain similar ratios of cell types across cell types. Cell types with lower sensitivity values in Tables 4 and 5 are expected to have lower prevalence in the automatic sets compared to the manual counterparts, whereas cell types with lower specificity are expected to have an increased prevalence in the automatic sets. Absolute numbers and relative amounts of each cell type are listed in Table 6 for both manual segmentations and automatic predictions for all three datasets.

**Table 6:** Cell counts for manual segmentations and automatic predictions in all datasets.

	Total cells (images)	Average cells/image	CD3+CD4+ T cells (%)	CD3+CD4− T cells (%)	mDCs (%)	pDCs (%)	B cells (%)
Manual							
Fresh-frozen-DS	5166 (240)	21.5	2688 (52.03)	1161 (22.48)	292 (5.65)	1025 (19.84)	N/A
FFPE-DS	7145 (160)	44.7	4104 (57.44)	2041 (28.57)	483 (6.76)	517 (7.23)	N/A
FFPE-SS	10,611 (342)	31.0	3714 (35.00)	2846 (26.82)	768 (7.24)	847 (7.98)	2436 (22.96)
Automatic							
Fresh-frozen-DS	16,666 (673)	24.8	8216 (49.30)	4047 (24.28)	2160 (12.96)	2243 (13.46)	N/A
FFPE-DS	16,396 (380)	43.1	8351 (50.93)	5340 (32.57)	1186 (7.23)	1519 (9.27)	N/A
FFPE-SS	38,594 (1332)	29.0	11126 (28.82)	14962 (38.76)	2573 (6.66)	2436 (6.31)	7506 (19.45)

### 3.2 Fixation Method Affects Cell Shape and Network Performance

**Fig. 5:**
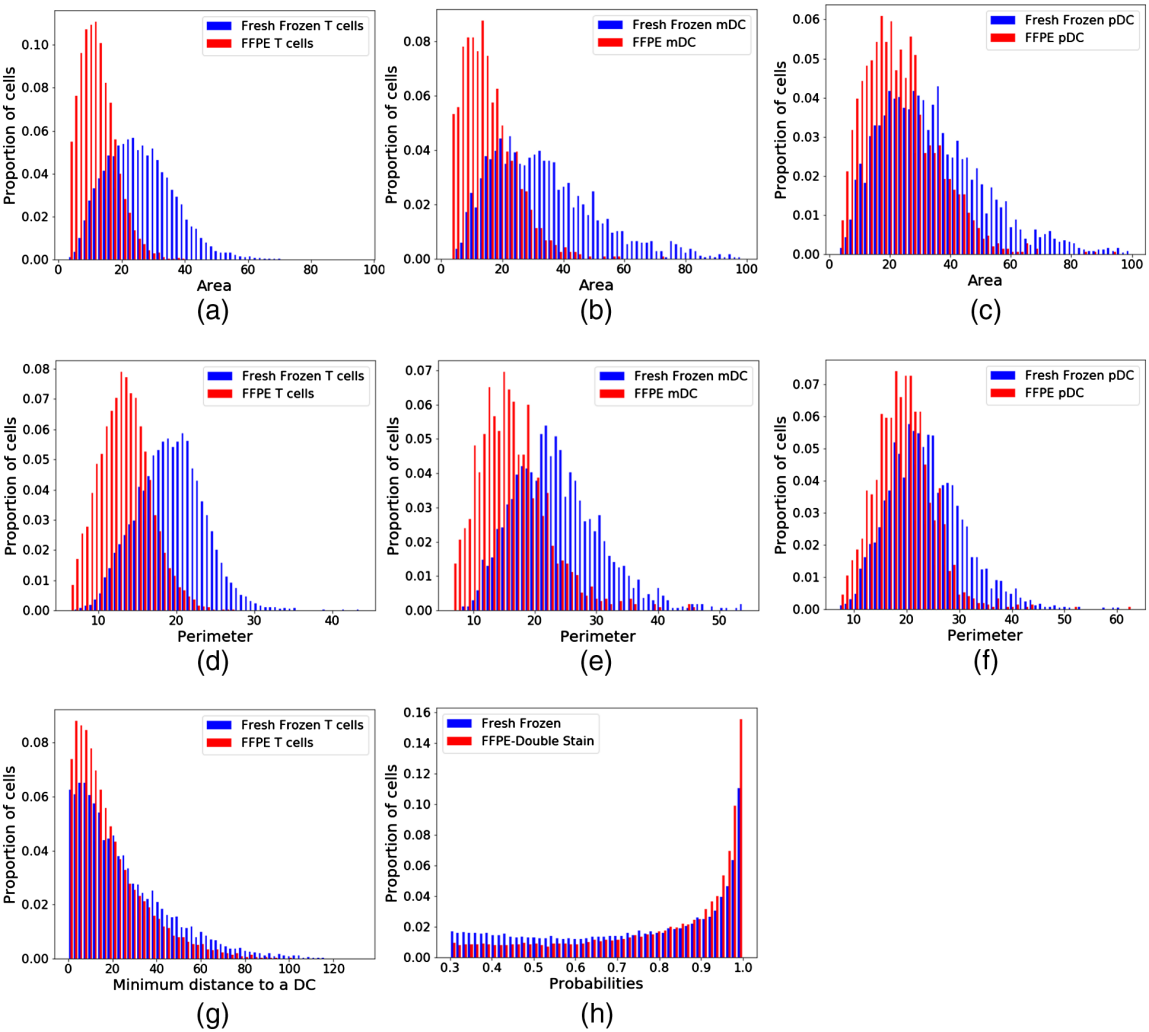
Shape and distance differences exist between cells of the same population when different fixation methods are used. Area of (a) T cells, (b) mDCs, and (c) pDCs is significantly smaller in FFPE samples than fresh-frozen samples. Perimeter of T (d) cells, (e) mDCs, and (f) pDCs is significantly smaller in FFPE samples than fresh-frozen samples. (g) The minimum distance between a T cell and the nearest DC is significantly smaller in FFPE than fresh-frozen samples. (h) Both networks show high confidence in the automatic predictions, although the FFPE probabilities are significantly higher. For all plots, a Kolmogorov–Smirnov test shows a statistical difference between distributions (p≪<0.0001).

It is widely documented that the processes of fresh freezing and formalin fixation cause different deformations to tissue. Formalin fixation will dehydrate the tissue, causing a contraction.^20^^–^^22^ Figures 5(a)–5(c) show that this phenomenon is consistent across all cell types. T cells and mDCs show a markedly reduced area in FFPE samples compared to their fresh-frozen counterparts [Figs. 5(a) and 5(b)]. However, while pDCs are also much smaller in FFPE than fresh-frozen samples, the change in area is less than that of T cells and mDCs [Fig. 5(c)]. In FFPE samples, pDCs showed a 31.4% reduction in mean area compared to 54.8% and 55.5% reductions in the mean area of mDCs and T cells, respectively. Similarly, a contraction of cellular perimeter was observed for all classes [Figs. 5(d)–5(f)]. This shrinkage is not only found at the cellular scale but remains consistent at the tissue level. Figure 5(g) shows the distribution of minimum distances of T cells to the nearest DC. T cells in FFPE samples show shorter distances to DCs than in fresh-frozen samples (p≪<0.0001). The fixation method therefore influences not only measurements of cell size and shape but of spatial relationships between cells. Both networks exhibited high confidence in the classifications, as measured by the distribution of probabilities assigned by the network, with the FFPE-DS network showing increased prediction probabilities relative to the fresh-frozen-DS network [Fig. 5(h)].

### 3.3 Staining Panel Affects Automated Detection of Cells

**Fig. 6:**
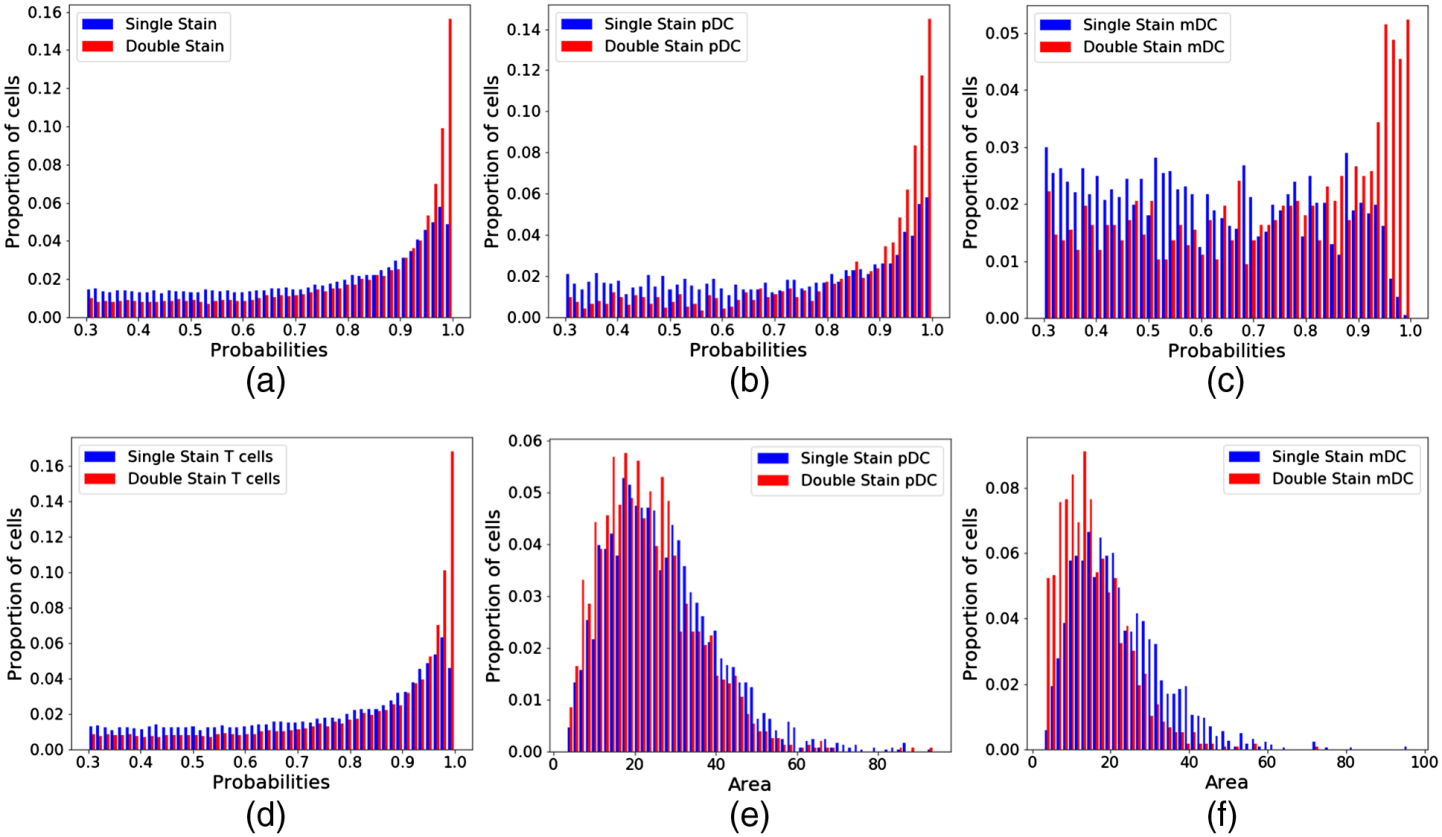
Number of stains used to probe a DC population affects the network performance. (a) Network confidence in cell classification for all cells is compared between a network trained on a single-stain DC panel and a network trained on a double-stain DC panel. The network trained on the double-stain panel was statistically more confident in its predictions (p≪<0.0001). (b) Probabilities of cells classified as pDCs by networks trained on single- and double-stain DC panels. (c) Probabilities of cells classified as mDCs by networks trained on single- and double-stain DC panels. (d) Probabilities of cells classified as either T cell population by networks trained on single- and double-stain DC panels. (a)–(d) Have a lower bound of 0.3 because cells below this threshold are automatically rejected by the network. (e) Cell area of pDCs detected by networks trained on single- and double-stain panels. (f) Cell area of mDCs detected by networks trained on single- and double-stain panels. For all plots in this figure, a Kolmogorov–Smirnov test shows a statistically significant difference between the two distributions (p≪<0.0001).

Separate staining panels were used on the two FFPE datasets to test the feasibility of using a single marker to identify APC populations. This would allow us to analyze a more diverse set of cells in a given biopsy, overcoming the technical limitations of antibody species and available microscope laser lines. For example, the FFPE-DS dataset can probe a single APC population—either mDCs or pDCs—in a given image, whereas the FFPE-SS dataset probes three APC populations—B cells, mDCs, and pDCs—in single image. The use of the single-stain system compared to the double-stain system diminished the accuracy of the network for DC populations (Fig. 6). Compared to the network trained on a panel with double-stained DCs, the network trained on the panel with single-stained DCs yielded worse confidence overall in cell detection and classification, as shown by the distribution of probability scores for the DC classes [Figs. 6(a)–6(c)]. This is consistent across all cell types, but particularly noticeable in mDCs [Fig. 6(c)], which corresponds with the poor sensitivity to mDCs with the network trained on the FFPE-SS dataset (Table 5). Furthermore, neither mDC nor pDC area remains consistent [Figs. 6(e) and 6(f)], suggesting that the decrease in sensitivity to these cells skews the distribution of cell features.

## 4 Discussion

Automated instance segmentation of LuN biopsies revealed quantifiable differences between cells and intercellular distances in fresh-frozen and FFPE biopsies. Additionally, staining panel design was found to affect the performance of automated instance segmentation of LuN biopsies with mask R-CNN.

### 4.1 Network Performance and Interpreting Observations from Unlabeled Datasets

Networks trained on samples stained with panel 1 had better overall sensitivity than samples stained with panel 2 (Tables 4 and 5), particularly for DCs. This indicates that a dual-marker system for identifying DCs is more effective for training an automatic cell detection and segmentation algorithm than a single-marker system. This is likely due to multiple factors. Having two markers for a given cell type provides a more stringent criteria for ground truth. Therefore, the ground truth for the double-stain DCs is less ambiguous, which translates into network performance. In generating ground truth, calling cells in the fresh-frozen samples was reported to be more difficult than calling cells in the FFPE samples, given the same staining panel. This likely contributes to the better overall sensitivity and specificity of an FFPE-trained network relative to fresh-frozen-trained network (Table 4).

In general, cell segmentation is particularly difficult in dense regions of cells. The three datasets interrogated in this paper have different average cell densities, ranging from 21 to 45 cells per image on average. The most densely packed dataset was the FFPE-DS dataset (45 cells per image). A network trained to segment cells in this dataset outperformed a network trained to segment cells in the least densely packed dataset, fresh-frozen-DS (21 cells per image) (Table 4). This further supports the notion that sample preparation affects the performance of these cell segmentation algorithms. The FFPE-SS dataset has an average of 31 cells per image compared to an average of 45 cells per image in the FFPE-DS dataset. T cells in these two datasets are stained with the same markers. There is a slight decrease in all performance metrics for a network trained on FFPE-DS images (most dense) compared to a network trained on FFPE-SS images (less dense) (Tables 4 and 5). These results suggest that the image quality variables associated with sample fixation, such as changes in tissue background and non-specific antibody binding, affect algorithm performance more so than cellular density.

The DC populations in the two DS datasets comprise a larger portion of the automatic cell predictions than manual (Table 6). This is because of the high sensitivity and moderate specificity (Table 4). Most DCs are detected, and false positives bolster the prevalence. Also, in the fresh-frozen-DS dataset, mDCs appear to increase in prevalence. However, this increase is due to a higher number of mDC images in the unlabeled fresh-frozen-DS dataset. While mDC images only comprised 30% of the ground truth set (training, validation, and testing), the unlabeled dataset was comprised of nearly 50% mDC images. Interestingly, in both FFPE datasets, the ratio CD3+CD4− T cells to CD3+CD4+ T cells increased in the automatic predictions (Table 6); however, the overall cell density per image remained fairly consistent between the manual and automatic segmentations (Table 6). This combined with the sensitivity and specificity for T cell populations in Tables 4 and 5 suggest that both FFPE-trained networks are misclassifying a fraction of CD3+CD4+ T cells as CD3+CD4− T cells. However, both of these networks detect and classify T cells well, with sensitivity and specificity values of 0.83 or greater (Tables 4 and 5).

Automatic predictions were done on larger unlabeled datasets to more effectively probe the ability of these networks to generalize to new images. Of the images that were manually segmented, a large portion (85% to 90%) was reserved for training, resulting in small validation and test sets. The test set for each dataset allows for a direct comparison of manual segmentations and automatic predictions. Comparing the density of cells detected per image and the relative numbers of each cell type (Table 6) in the manual segmentations and automatic predictions further shows the generalizability of these networks to new data without requiring hundreds of more image images and thousands of more cells to be manually segmented.

### 4.2 Network Generalizability to other Tissue Fixation Methods

Because the fresh-frozen-DS and FFPE-DS datasets have the same number of channels and the same number of classes, it is possible to test the generalizability of a network trained on one dataset by using it to generate predictions on the other. Additionally, we can observe the differences in training a network on “hard examples” compared to “easier examples.” For this staining panel, tissue fixed with the fresh-frozen method was reported to have more ambiguous cells by the experts who collected the data and provided ground truth, making this a hard example training set, whereas the FFPE samples were clearer, making this an easier example training set. The network trained on the fresh-frozen-DS dataset was used to make predictions on the FFPE-DS test set and vice versa. Both networks generalized fairly well to the new test sets (Table 7). Interestingly, neither network generalized better than the other across all cell types. For example, the network trained on FFPE samples generalized better to DCs in fresh-frozen samples, whereas the network trained on fresh-frozen samples generalized better to CD3+CD4+ T cells in FFPE samples. In general, the trends in sensitivity, specificity, and IOU follow the trends in Table 4, where each network was tested on data from the same fixation method. This shows that these methods for detecting cells in biopsies can generalize to images of samples with different fixation methods and images with different pixel size/resolution. However, if a network was intended to be used to detect and classify cells in biopsies from multiple fixation methods, and/or in images variable pixel size, and this was known prior to training, more consistent performance would come from training the network on a merged set of ground truth images.

**Table 7:** Network performance for a network trained on fresh-frozen samples, but tested on FFPE samples, and vice versa.

	CD3+CD4+ T cells	CD3+CD4− T cells	DCs	All cells (average)
Sensitivity				
Train on fresh frozen, test on FFPE	0.88	0.68	0.83	0.80
Train on FFPE, test on fresh frozen	0.74	0.78	0.94	0.82
Specificity				
Train on fresh frozen, test on FFPE	0.81	0.84	0.91	0.85
Train on FFPE, test on fresh frozen	0.66	0.70	0.93	0.76
IOU				
Train on fresh frozen, test on FFPE	0.76±0.21	0.69±0.25	0.79±0.23	0.73±0.23
Train on FFPE, test on fresh frozen	0.80±0.19	0.76±0.25	0.81±0.18	0.80±0.20

### 4.3 Implications of Variable Cell Shape across Tissue Fixation Methods

Figure 5 demonstrates that tissue fixation impacts the metrics of cell shape and intracellular distances that can be derived from the network predictions. Tissue expansion and shrinkage in fresh-frozen and FFPE tissue, respectively, is well-documented.^20^^–^^22^ The data presented here quantify these deformations, showing a ∼30% decrease in all linear metrics of T cell shape (e.g., equivalent diameter and perimeter), a 52.7% decrease in mean T cell area, and a 24.7% decrease in the minimum distance of a T cell to the nearest DC. These discrepancies in cellular features can have implications on conclusions drawn from data mining images to investigate biological phenomena. Previous work has used cellular shape and distance between cell types in fresh-frozen LuN biopsies to identify intercellular interactions.^10^ Metrics including minimum distance of T cells to a DC and T cell shape features were used to identify which cell populations were more frequently interacting. For this work to translate effectively to FFPE LuN biopsies, these differences in cell size, cell shape, and intercellular distances must be taken into consideration.

### 4.4 Multiple Markers for Classification

The second major technical consideration we investigated is the utility of using multiple markers for classifying cells, particularly for difficult classes such as dendritic cells. Because a given immunofluorescence experiment is limited to 5 to 6 markers, there is a real cost associated with using multiple markers per cell type. In panel design, there is a trade-off between robustly identifying a single cell type and interrogating multiple cell types in a single experiment. We evaluated the extent to which using a single stain to identify DC subsets diminished network performance. We observed that the network sensitivity was relatively poor for the single-stain dataset, particularly for mDCs and pDCs. DC subsets were particularly impacted by ambiguous staining from single markers, compounded by relatively low prevalence of these cell types in the dataset. This loss of sensitivity had consequences for calculating cell features downstream, as evidenced by the shift in the observed area distribution for pDCs and mDCs [Figs. 6(e) and 6(f)]. Thus, we conclude that using a single marker for detecting difficult or infrequent cell types is not a worthwhile compromise, because the benefit of interrogating multiple cell types is negated by the decrease of algorithm robustness in detecting these infrequent cell types. Using multiple markers will bolster the performance of computer detection of cells, particularly for cell classes of lower prevalence. Because of a severe class imbalance with DCs in the underlying biology of LuN, it is imperative that we optimize sample staining to ensure adequate instance segmentation of these cells.

## 5 Conclusions

Three separate instances of a mask R-CNN architecture were trained on three datasets of fluorescence confocal images of LuN biopsies in order to evaluate which elements of data collection can drive the success of computer vision-based analytical approaches. Automatic segmentation of these datasets confirms that fixation method of the tissue affects cell shape features and intercellular distances. Specifically, these features are quantifiably smaller in FFPE samples compared to their fresh-frozen counterparts. Additionally, we demonstrated that using multiple markers to delineate difficult cell classes is essential to optimize automated detection of cells in LuN biopsies. These data show that decisions around tissue preparation and marker panels are important factors to consider and optimize in order to extract biologically relevant information from clinical biopsies.

## Code, Data, and Materials Availability

The general mask R-CNN code used in this paper can be found online at https://github.com/matterport/Mask_RCNN. The specific image datasets and code used for downstream analysis can be made available upon request.
