# Supplementary material for: Quantifying the effects of biopsy fixation and staining panel design on automatic instance segmentation of immune cells in human lupus nephritis
Source: J Biomed Opt. 2021 Jan 8;26(2):022910. doi: 10.1117/1.JBO.26.2.022910 (PMC7791891; doi:10.1117/1.JBO.26.2.022910)
Supplement: Supplementary file 1 [file JBO_026_022910_SD001.pdf]

Supplementary Information

Antibody staining

Table S1 shows the details for selected antibodies for both fresh frozen and FFPE samples. A single panel was used on fresh frozen samples. In this work, two FFPE datasets were used, each with different staining. Samples in the FFPE-DS dataset were stained for T cells and one dendritic cell population: either myeloid dendritic cells (mDCs) or plasmacytoid dendritic cells (pDCs). The mDC panel for the FFPE-DS dataset consisted of: CD3, CD4, BDCA1, and CD11c. The pDC panel for the FFPE-DS dataset consisted of: CD3, CD4, BDCA2, and CD123. Samples in the FFPE-SS dataset were stained for T cells, B cells, pDCs, and mDCs. The FFPE-SS panel consisted of: CD3, CD4, CD20, BDCA2, and CD11c.

Table S1. Details on selected antibodies used for staining of fresh frozen and FFPE samples.

Primary antibodies for fresh frozen sections

Antibody target	**Clone**	**Vendor**	**Catalog #**	**Dilution**
CD3	SP7	abcam	ab16669	1:100
	CD3-12	AbD Serotec	MCA1477F	1:50
CD4	YNB46.1.8	abcam	ab34276	1:100
CD303 (BDCA2)	AC144	Miltenyi Biotec	130-090-510	1:10
CD11c	EP1347Y	abcam	ab52632	1:100
CD1c (BDCA1)	L161	Beckman Coulter	IM0789	1:100
CD123	6H6	eBioscience	14-1239-82	1:100

Primary antibodies for FFPE sections

Antibody target	**Clone**	**Vendor**	**Catalog #**	**Dilution**
BDCA2	polyclonal goat IgG	R&D Systems	AF1376	1:50
CD4	EPR6855	abcam	ab196147	1:100
CD3	CD3-12	AbD Serotec	MCA1477	1:100
CD1c (BDCA1)	L161	Beckman Coulter	IM0789	1:100
CD123	6H6	eBioscience	14-1239-82	1:100
CD11c	EP1347Y	abcam	ab52632	1:100
CD20	L26	Agilent	M0755	1:100
